# Large language model-assisted modern programming pedagogy model: a perspective on teaching programming in the era of LLMs

**DOI:** 10.3389/frai.2026.1945247

**Published:** 2026-10-28

**Authors:** Rex Bringula, Fredrik Heintz

**Affiliations:** Linköping University, Linköping, Sweden

**Keywords:** AI tools, educational theories, generative AI, programming education, programming learning

## Abstract

Large language models (LLMs) are a double-edged technology. They can serve as learning assistants in programming while simultaneously raising pedagogical and ethical concerns. Programming education researchers have responded by providing guidelines and recommendations on teaching, assessment, and the ethical use of LLMs. Although these developments are welcome, the literature still lacks theory-based frameworks that provide a philosophical grounding for programming pedagogy. This perspective paper addresses this gap by proposing the LLM-Assisted Modern Programming Pedagogy (LAMPP) model. The model aims to provide a theory-informed framework for systematically guiding the integration of LLMs into programming pedagogy. It proposes four design principles encompassing the core components of pedagogy (i.e., teaching, lesson design, engagement, and assessment). Ethics is included as an additional dimension because ethical concerns cut across all aspects of pedagogy. The LAMPP model proposes different mechanisms that may mitigate potential learning losses associated with inappropriate or excessive reliance on LLMs. Future studies should test the effectiveness of these mechanisms. The model may provide a basis for educators, researchers, and policymakers when considering policies, curriculum structures, course outcomes, assessment practices, and ethical guidelines related to programming pedagogy. In summary, it is not about LLM tools themselves but about the pedagogical orchestration of these technologies in programming education.

## 1 Introduction

Programming is a difficult task. It requires exceptional accuracy because it integrates procedural and conceptual knowledge in a precise, logical way (McCracken et al., 2001; Lister et al., 2004). Before the introduction of large language models (LLMs; hereafter, AI tools), students coded independently or collaborated with peers. They need to master problem-solving strategies, algorithm development, programming-language syntax, debugging, testing, and code optimization (Bailey and Mitchell, 2006). Students often get information from lectures, textbooks, YouTube videos, programmers’ blogs, and other educational materials (Lausa et al., 2021). Students debug manually, with feedback from teachers, classmates, or professional programmers (Michaeli and Romeike, 2019). In other words, developing working software depends on students’ use of these resources, debugging abilities, and their overall programming competence (Bailey and Mitchell, 2006).

Teaching programming is typically teacher-led, with teachers explaining problem-solving strategies, programming syntax, the programming environment, and algorithms. They generally prepared educational materials manually by designing lecture slides, coding examples, assignments, and exams. These materials often drew on textbooks, personal expertise, and other educational resources. They frequently reused or adapted resources from previous courses and open educational materials, refining them over time based on student performance and common difficulties (Salas, 2020). The process was largely labor-intensive and depended heavily on the teachers’ experience and judgment.

Before the introduction of LLMs, several studies reported different pedagogical approaches to improve students’ learning, engagement, and programming performance. Vihavainen et al. (2014) found that cooperative learning among students (e.g., pair programming, peer support, collaboration) improved programming performance. In a systematic review of 24 studies selected from 2012 to 2018, the four main teaching approaches were cognitive apprenticeship, problem-based learning, robots, and miscellaneous. Robots refer to the use of LEGO technology. These approaches resulted in high student satisfaction, motivation, engagement, and retention (Santos et al., 2020). Moreover, these approaches fostered the development of technical and soft skills (Santos et al., 2020). In a similar study, 40 papers published between 2014 and 2019 on the different pedagogical techniques of active learning in the content of teaching and learning computer programming, the most prominent strategies were the flipped classroom (*n* = 17), project-based learning (*n* = 6), and peer instruction (*n* = 4). The major barrier to using active learning pedagogical techniques is that they require more effort from teachers to plan and implement (Berssanette and de Francisco, 2021).

Instructional practices were complemented by structured assessments designed to determine whether students had acquired the necessary conceptual and procedural knowledge. Teachers conduct structured assessments of conceptual (i.e., designed to measure understanding of programming principles rather than coding execution skills) and procedural knowledge (i.e., students can execute steps, apply rules, and produce working code rather than just explain concepts; (McCormick, 1997). The former can be in the form of structured tools (e.g., test, inventory, rubric, or interview protocol), while the latter can be in the form of a live coding test, take-home machine problem test, practical programming exam, or debugging task, among others (Daly and Waldron, 2004).

Other studies suggested a range of formats designed to capture different aspects of students’ programming learning. These assessment formats included selected-response assessments (e.g., multiple-selection, multiple-choice, single-choice, true/false, and assertion–reason items), code-based assessments (e.g., developing code from scratch, predicting program output, and code-based fill-in-the-blank tasks), performance-based assessments (e.g., interviews and project portfolios), and peer-based assessment (e.g., peer review; Brennan and Resnick, 2012; Gomes et al., 2016; Sun et al., 2019). Furthermore, programming educational outcomes included coding skills, problem-solving, critical thinking, social skills, and academic skills (excluding mathematical or computer-related skills; Popat and Starkey, 2019). These educational outcomes were measured through pre- and post-assessments of standardized tests, comparison tests of reflective thinking skills and problem-solving, teacher-made pretests and posttests, observations, and other qualitative data collection (Popat and Starkey, 2019).

The emergence of LLMs has disrupted the established approaches to teaching and learning programming. Teachers can utilize LLMs to generate instructional materials, coding examples, and assessment items (Bringula, 2024). Students, meanwhile, use these AI tools to support their understanding of programming concepts, generate code, debug programs, test outputs, and optimize code (Bringula, 2024). Students can now produce correct or near-correct code instantly without going through the traditional learning process (Alquran and Banitaan, 2026). Consequently, programming becomes less challenging (Alquran and Banitaan, 2026).

These shifts create tensions and raise ethical concerns that challenge the core foundations of programming pedagogy (e.g., teaching strategies, lesson design, facilitation of student engagement, and assessment). The sudden shift from a process-oriented to a solution-oriented approach creates tension in teaching programming. Students risk underdeveloping algorithmic thinking if they become overly dependent on instant, polished code (Király and Troll, 2025). Hence, there is a pressing need to develop teaching strategies and authentic tasks that encourage creativity and exploration. Teachers must reconsider how they structure activities and learning environments to promote meaningful learning.

Another tension arises in balancing students’ involvement in programming with the use of AI tools. Teachers must ensure students do not turn to AI tools too quickly and instead instill the value of productive struggle and perseverance (Jose et al., 2025; Wiederhold, 2025). LLM-generated outputs make it more difficult for teachers to assess students’ understanding based solely on final code outputs. When students submit code that is partially or fully LLM-generated, it becomes difficult to determine what they truly understand. This threatens the validity and reliability of traditional assessment methods (e.g., take-home machine problems). This raises another issue for authentic skill assessment methods.

Conceptual knowledge assessment is also affected because AI tools can generate correct responses to text-based assessments (e.g., tests; Susnjak and McIntosh, 2024), which is often considered cheating. These situations create tensions between AI tool assistance and academic integrity, productivity and conceptual understanding, personalization and over-reliance, and meaningful engagement and passive use. These tensions also raise ethical questions about responsible LLM use in programming education.

## 2 The development of LLM-assisted modern programming pedagogy (LAMPP) model

Several studies partially address the pedagogical challenges of AI tools in programming education (Azemi, 2025; Gəruslu et al., 2026; Liang, 2026). Researchers have not thoroughly explored the full components of pedagogy (i.e., teaching, lesson design, engagement, and assessment) in relation to LLMs. Moreover, education theory–driven design frameworks for these technologies remain underdeveloped (Fu et al., 2025). These gaps suggest revisiting and reforming these four pedagogical components by including ethics as a cross-cutting dimension. Findings from previous studies provide the basis for identifying how each component needs reconsideration in the LLM era (Table 1), which then informs the model’s four design principles.

**Table 1:** Theoretical and literature-based rationale for the LAMPP components.

LAMPP components	LLM-related pedagogical challenges	References	Theoretical basis
Teaching	LLMs transform teachers’ roles in LLM-facilitated learning environments.	Aissa and Mhiri (2025), Aruleba et al. (2025), Azemi (2025), Fu et al. (2025), Gəruslu et al. (2026), Haruto et al. (2025), Király and Troll (2025), Mahon et al. (2024), Raihan et al. (2025) and Stoyanova et al. (2025)	Constructivism Social constructivism Cognitive apprenticeship
Lesson design	LLMs can generate or complete programming tasks, disrupting conventional lesson design.	Aruleba et al. (2025), Azemi (2025), Fu et al. (2025), Gəruslu et al. (2026), Haruto et al. (2025), Király and Troll (2025), Mahon et al. (2024), Raihan et al. (2025) and Stoyanova et al. (2025)	Self-regulated learning Social constructivism
Learning engagement	LLMs change how students interact with programming problems, feedback, and learning resources.	Azemi (2025), Aruleba et al. (2025), Gəruslu et al. (2026), Haruto et al. (2025), Király and Troll (2025) and Zhu et al. (2026)	Constructivism Self-regulated learning
Assessment	LLMs can generate programming solutions and test answers, challenging conventional assessment.	Azemi (2025), Gəruslu et al. (2026), Király and Troll (2025), Liang (2026), Mahon et al. (2024), Raihan et al. (2025) and Stoyanova et al. (2025)	Constructivism Social constructivism Self-regulated learning Cognitive apprenticeship
Ethics	LLMs create issues involving academic integrity, responsible use, transparency, digital divide, etc.	Aruleba et al. (2025), Fu et al. (2025), Gəruslu et al. (2026), Jiao et al. (2025), Király and Troll (2025), Kizilcec et al. (2024), Mahon et al. (2024) and Stoyanova et al. (2025)	Social constructivism Self-regulated learning

Studies have found that LLMs are shifting teachers from traditional code instructors to facilitators and designers of AI-supported learning environments (Fu et al., 2025; Mahon et al., 2024; Raihan et al., 2025). Other studies also emphasized that teachers need to provide structured guidance and oversight to prevent students from relying too heavily and passively on these technologies (Király and Troll, 2025; Haruto et al., 2025). Both teachers and students viewed them primarily as supplementary tools that can facilitate personalized learning and provide additional instructional support (Fu et al., 2025; Azemi, 2025). Nonetheless, teachers remain essential in the era of LLMs because they must ensure students develop critical thinking, code evaluation, problem-solving, and algorithmic thinking skills rather than focusing on code production alone (Stoyanova et al., 2025). Teachers’ roles may need to adapt to the changing programming education landscape. They are expected to facilitate, guide, and oversee learning in an LLM-assisted environment. Hence, LLM-facilitated learning calls for reconsidering existing teaching practices.

Teachers’ changing roles also require a corresponding transformation in programming lesson and learning-activity design. Several studies agree that lesson design should incorporate structured activities, such as self-assessment, validation, peer review, and code comparison (Aruleba et al., 2025). These activities promote active engagement and reduce passive reliance on LLMs (Király and Troll, 2025). Lessons must also engage students in critical use of AI tools. Teachers can achieve this through activities such as code comprehension, code explanation, and validation (Gəruslu et al., 2026; Azemi, 2025). Another important skill teachers need to develop in students is prompt engineering, which helps students communicate their intended outputs more effectively with AI tools (Haruto et al., 2025). Thus, learning activities should deliberately incorporate these technologies while preserving intended learning objectives.

Teachers consistently reported overreliance and passive reliance as two major concerns in an LLM-assisted learning environment (Azemi, 2025; Fu et al., 2025; Haruto et al., 2025). Students may rely heavily on LLMs for code generation, problem-solving, and completing programming tasks (Aruleba et al., 2025; Raihan et al., 2025). On the other hand, they may also use LLMs as interactive learning companions that explain code and concepts, assist with debugging, and provide feedback (Fu et al., 2025; Haruto et al., 2025). Azemi (2025) further shows that LLMs may provide multilingual support, increase students’ confidence, reduce frustration, and promote inclusive participation. Thus, evidence shows that students engage with LLMs in different ways, ranging from meaningful engagement to passive dependence. Therefore, it is important to cultivate meaningful engagement further while gradually reducing dependence on AI.

Another important issue raised in the literature is how to accurately and fairly assess students’ programming competence in the era of LLMs (Azemi, 2025). LLMs profoundly affect conventional assessment because they can generate, complete, and improve programming solutions. These capabilities make traditional assessment and AI-detection methods less reliable (Elkhatat et al., 2023; Nikolopoulou, 2025). Consequently, teachers may struggle to determine whether submitted work reflects students’ actual programming competence (Stoyanova et al., 2025; Raihan et al., 2025). Assessment methods therefore need to shift from evaluating final outputs toward process-oriented skills (Gəruslu et al., 2026). To support this shift, Stoyanova et al. (2025) and Mahon et al. (2024) suggest supervised examinations, oral defenses, group presentations, and project-based assessments.

The challenges LLMs introduce across these four pedagogical components also raise ethical concerns. Teachers are at the forefront of instilling responsible AI use. They are expected to guide students toward appropriate, transparent, and responsible LLM use (Mahon et al., 2024). They are encouraged to address potential excessive LLM use, plagiarism, cheating, unauthorized AI use, and students' attempts to bypass AI detection (Stoyanova et al., 2025; Fu et al., 2025). They are also expected to help students recognize and critically evaluate inaccurate, biased, or misleading LLM outputs (Aruleba et al., 2025; Jiao et al., 2025). They should also recognize potential inequalities in access to the technology (Fu et al., 2025; Kizilcec et al., 2024).

Of the 14 relevant studies reviewed, five empirical studies used theories to explain LLM use in programming education. Aissa and Mhiri (2025) used constructivism, Piaget’s theory of cognitive development, and personal construct theory to evaluate ChatGPT’s effectiveness in improving programming skills and logical thinking among second-year Tunisian computer science students. Constructivism viewed learning Python with ChatGPT as an active process where students construct knowledge through interaction, code execution, and personalized feedback. Piaget’s theory of cognitive development explained how students assimilate and accommodate new programming concepts based on their prior knowledge. Personal construct theory provided the foundation for how students perceive, interpret, and make sense of LLMs.

Azemi (2025) explored how ChatGPT and GitHub Copilot have transformed education by shifting the pedagogical focus toward design, systems thinking, and ethical reasoning. The study used Cognitive Load Theory (CLT) and the Ethics of Care as its framework. CLT suggests that AI can offload the cognitive load of generating low-level coding syntax. This allowed students to focus on algorithms, logic, and problem decomposition. The Ethics of Care guided the formulation of AI-use policies, assessment, and academic-integrity practices.

Gəruslu et al. (2026) also used constructivism in their study. They also used sociotechnical systems thinking because the study focused on understanding the relationships among teachers, students, and AI tools within the learning system. Moreover, they used Bloom's taxonomy as an assessment framework for students’ learning activities. In another study, Zhu et al. (2026) used self-regulated learning to determine undergraduate students’ learning outcomes in a 16-week programming course. The study found significant gains in students’ programming learning. However, the study did not compare these findings with alternative instructional conditions (e.g., a group that did not use LLMs).

In a recent study, an LLM was viewed as a human expert that assisted students in programming (Liang, 2026). The system design followed cognitive apprenticeship theory (Collins et al., 1991). The system incorporated an LLM to analyze students’ programming work, identify learning difficulties, and provide support. Teachers also provided support alongside LLM assistance. The study showed that students’ programming performance improved. However, Liang (2026) noted that causal inference was limited because the study employed only a single-group research design.

The previously discussed studies and theoretical perspectives provide valuable insights into different aspects of LLM-facilitated learning environments. Building on these existing studies and theoretical insights, this perspective study proposes the LLM-Assisted Modern Programming Pedagogy (LAMPP) Model (Figure 1). The LAMPP Model (hereafter, the model) provides a holistic, theory-grounded framework that guides educators in systematically integrating LLMs into programming pedagogy while preserving core learning goals such as problem-solving, conceptual understanding, and academic integrity.

**Figure 1:**
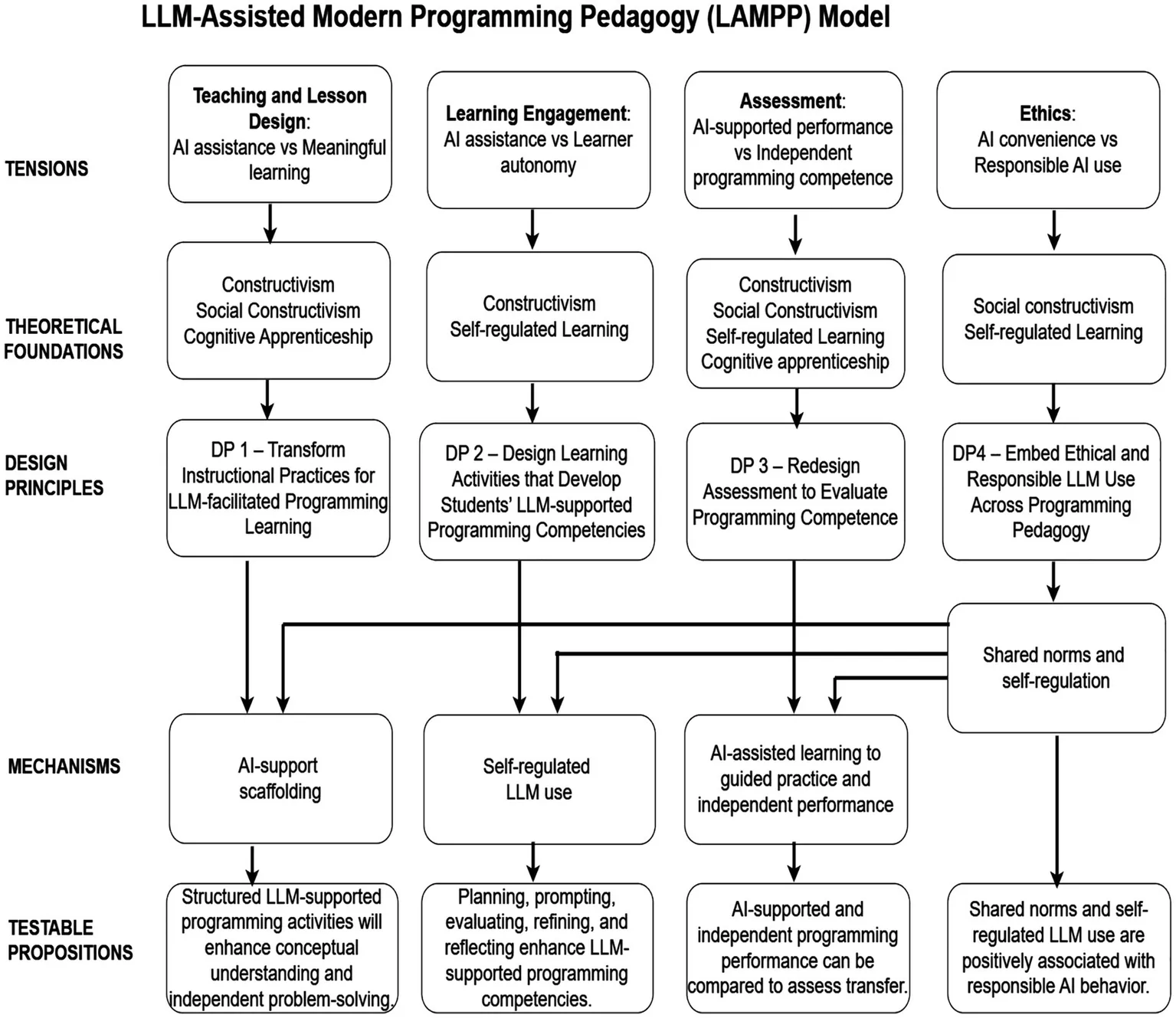
The LLM-assisted modern programming pedagogy (LAMPP) model.

Unlike prior work that has focused on isolated interventions (e.g., using LLMs for tutoring or code generation; Martini, 2024; Vukojičić and Krstić, 2023), this study identifies the tensions LLMs introduce into programming pedagogy. The model then aims to align teaching strategies, lesson design, student learning engagement, assessment practices, and ethical considerations within a coherent, theory-based framework. It bridges emerging AI tools and established learning theories, including constructivism, social constructivism, self-regulated learning, and cognitive apprenticeship. The four identified tensions provide compelling reasons to develop a framework for programming pedagogy in response to the disruptions LLMs bring to programming education. The study mapped each tension to educational theories to ground educational responses and justify these programming pedagogical changes.

The LAMPP model’s central premise is that LLMs are valuable learning tools, but teachers should integrate them carefully into programming pedagogy. Teachers are viewed as facilitators of LLM use in the classroom, while students create their own learning with LLM support. Students are allowed to use LLMs because these technologies can act as coding assistants or expert-like resources. However, students must actively regulate their LLM use by deciding when to seek assistance, evaluating the responses they receive, and reflecting on how the interaction contributes to their learning. Constructivism, social constructivism, cognitive apprenticeship, and self-regulated learning (SRL) align well with these assumptions and could offer guiding perspectives for addressing the tensions discussed earlier.

Consistent with the view that learners actively construct knowledge through experience and reflection rather than passively receiving information, constructivism provides the foundational perspective for the LAMPP model. This theory is relevant because it discourages using LLMs for automatic code generation, which can lead to a lack of understanding of how or why the code works. From a constructivist perspective, LLM use should therefore be integrated into activities that require students to formulate solutions, test ideas, interpret outputs, and reflect on their understanding. This allows students to leverage LLM assistance, but it has to be structured in ways that preserve students’ active role in constructing programming knowledge. This selection is consistent with theoretical perspectives in previous studies (Aissa and Mhiri, 2025; Bada, 2015).

The LAMPP model views LLM interaction as a learning resource that can help students become more independent. Social constructivism is aligned well with this view because learning occurs through interaction and knowledge exchange among students, teachers, and LLMs (Adams, 2006). In this context, students do not simply receive information from LLMs; instead, they interact with LLMs by asking questions, evaluating generated responses, refining their prompts, testing and debugging code, and incorporating or rejecting AI-generated suggestions based on their own understanding. This interaction can support the social construction of knowledge, in which dialog, feedback, and the learner’s engagement with AI tools shape learning. Social constructivism is more appropriate than sociotechnical systems thinking because the former focuses on how learners construct knowledge through interactions with LLMs. In contrast, the latter focuses on the interactions between technology and organizations.

Another theory relevant to the study's assumptions is Self-Regulated Learning (SRL) theory. This theory addresses the tensions in learning engagement, assessment, and ethics. It can potentially address the issue in learning engagement because students should take greater control of their learning by setting goals, monitoring their progress, evaluating their understanding, and adjusting their learning strategies to achieve desired outcomes (Zimmerman and Schunk, 2011). The theory may support assessment practices because it provides a basis for evaluating not only the final code but also how students use and learn from the LLMs. Moreover, it may also address ethical concerns because students can regulate their usage behavior in line with shared classroom norms. In other words, this theory may reinforce the model’s emphasis on students as proactive, responsible learners. SRL aligns more closely than self-determination theory (Ryan and Deci, 2022) because SRL can explain how students actively regulate and evaluate their learning with LLMs, while SDT explains the motivational conditions that support learning.

Finally, Cognitive Apprenticeship Theory supports the model’s assumptions because LLMs can function as learning assistants (Liang, 2026). Similar to an expert or mentor, LLMs can provide solutions, scaffolding, and feedback. These technologies may take on different roles (e.g., coding assistant, explainer, debugger, coach, or feedback provider). After receiving assistance from a learning assistant, researchers can evaluate students’ programming performance. These perspectives align with the cognitive apprenticeship theory (Collins et al., 1991). Within the LAMPP model, the LLM is conceptualized not as a code generator but as an educational tool whose role can be deliberately designed around the learning objective set by the teacher. Overall, these theories were selected because they align with the model’s central theme and underlying assumptions, address distinct but interconnected dimensions of LLM-supported pedagogy, and are relevant to the pedagogical orchestration of LLMs.

These educational theories formed the basis for the design principles that operationalize how programming pedagogy could be reformed. Specifically, the model consists of the following four broad design principles:

1. Design Principle 1—Transform Instructional Practices for LLM-facilitated Programming Learning,
2. Design Principle 2—Design Learning Activities that Develop Students’ LLM-supported Programming Competencies,
3. Design Principle 3—Redesign Assessment to Evaluate Programming Competence, and
4. Design Principle 4—Embed Ethical and Responsible LLM Use Across Programming Pedagogy.

The mechanisms then show how the design principles translate into actionable programming pedagogies. Shared norms guided all other mechanisms. The study also proposes testable hypotheses to assess the model’s effectiveness.

## 3 Theory-informed discussion of design principles of the LAMPP model

### 3.1 Design principle 1—transform instructional practices for LLM-facilitated programming learning (DP1)

Design Principle 1 primarily focuses on teaching strategies and lesson design. The model does not eliminate teacher-centered scaffolding of students’ learning processes; instead, it augments such scaffolding through structured interactions with LLMs. Guided by constructivism, the model prioritizes active learning, with teachers engaging students in problem-solving rather than allowing them to rely on LLM-generated results passively (Bada, 2015). Teachers are encouraged to design lessons that intentionally incorporate LLMs as tools for exploration, comparison, feedback, and refinement rather than as direct providers of solutions.

This design principle centers on AI-supported scaffolding followed by students’ interpretation, testing, and refinement. Students remain responsible for interpreting AI outputs and constructing their solutions. This process addresses the tension between AI assistance and students’ meaningful learning. For example, teachers may provide students with a partially working program to debug. Students can seek assistance from an LLM by requesting hints or explanations of errors rather than complete solutions. They then formulate their own solutions based on these explanations, revise the code, run it, test different inputs, observe the outputs, and gradually refine the program. Through this process, the teacher uses the LLM to scaffold reflection and deeper analysis, while students remain responsible for understanding the programming problem and developing the final solution. The effectiveness of this design principle can be examined empirically through students’ conceptual understanding, debugging performance, explanation quality, and ability to solve related programming problems independently. This could potentially address the gap identified in the literature regarding students’ limited conceptual understanding of programming (Alanazi et al., 2025).

Social constructivism and cognitive apprenticeship are two theories that support using LLMs as learning aids (Adams, 2006). From a social constructivist perspective, LLMs could serve as scaffolding agents and could foster student–AI collaboration within the students’ zone of proximal development (Mishra, 2023). Meanwhile, cognitive apprenticeship posits that apprentices (i.e., students) can learn from experts (i.e., LLMs), who provide feedback, hints, reminders, and other forms of guidance (Shuell, 2001). The main process involves an iterative cycle in which students formulate solutions, receive feedback from LLMs, evaluate it, and refine their work. However, this must be structured to ensure meaningful interaction. For example, students may first construct an algorithm independently, then use an LLM to get feedback. They then evaluate the feedback, revise their algorithm, and repeat the process until they arrive at a satisfactory solution. Thus, LLMs serve to extend students’ problem-solving processes rather than replace them. This mechanism may help alleviate the tension between AI assistance and learner autonomy. Empirical data can test whether planning, prompting, evaluation, refinement, and reflection enhance students’ LLM-supported programming competencies. One recent study has already tested how self-regulated learning influenced programming learning (Zhu et al., 2026). However, it remains unclear which shared norms were agreed upon in that learning context and how these norms influenced the findings of the study.

### 3.2 Design principle 2—design learning activities that develop students’ LLM-supported programming competencies (DP2)

AI tools can help students code. This shifts agency from purely human code production to LLM-assisted programming. This creates tension between learner autonomy and AI assistance. The second design principle of the LAMPP model may address this issue. The model perceives these technologies as collaborators or scaffolding partners. In addition to established skill sets required to write functional programs (e.g., problem solving, coding, modifying, debugging, testing, and optimizing code; Bailey and Mitchell, 2006), there is a need to identify additional technical knowledge, skills, and competencies required to effectively use AI tools while still ensuring the development of students’ programming abilities. This design principle aligns with the model’s student engagement and lesson design components. Constructivist learning theory provides a foundation for addressing this question. It emphasizes developing strong conceptual understanding to interpret and evaluate LLM-generated outputs. Furthermore, lessons should include activities that require explanation, comparison, and reasoning. These activities might discourage passive acceptance of AI-generated outputs.

Similarly, self-regulated learning (SRL) emphasizes meaningful student engagement (Zimmerman and Schunk, 2011). This includes students planning how to use AI tools, evaluating output accuracy, and determining its relevance to tasks. Social constructivism further frames LLMs as scaffolding partners that function as interactive collaborators, providing explanations and feedback. Within this perspective, students must clearly communicate their intended outputs and refine iteratively until they achieve solutions.

DP2 focuses on developing competencies needed to program effectively with LLMs. The core mechanism includes planning, prompting, evaluation, refinement, and reflection. Through this process, students formulate intended solutions, communicate effectively with AI tools, critically evaluate outputs, revise solutions, and reflect on their learning process. Instructional activities should be carefully designed to promote active, reflective engagement and clearly articulated intent when interacting with AI tools. These activities should develop competencies such as critical thinking, metacognitive skills, prompt formulation, communication, output evaluation, and reflection.

The proposed mechanism can be empirically examined through measures such as prompt quality, evaluation tasks, reflective activities, and programming performance. Future studies may determine whether students who engage in LLM-supported programming activities that require them to plan their AI use, formulate prompts, evaluate LLM outputs, refine their solutions, and reflect on the process will demonstrate higher levels of LLM-supported programming competencies than those who primarily use LLMs to generate code or those who do not use LLMs at all.

### 3.3 Design principle 3—redesign assessment to evaluate programming competence (DP3)

LLMs have affected both procedural and conceptual knowledge assessments. LLMs can generate accurate responses to text-based questions; that is, text-based examinations administered through online platforms can potentially be answered using LLMs (Marco-Detchart and Lopez-Molina, 2025). Machine problem assignments, in which students must construct programs, are also affected because LLMs can generate code. These situations raise concerns about the integrity and reliability of assessment methods. Thus, a clear tension emerges between AI-supported performance and independent programming competence.

DP3 addresses this tension by emphasizing assessment approaches that evaluate not only the final code but also students’ reasoning, decision-making, and ability to apply programming knowledge independently. Constructivism and self-regulated learning support assessment approaches that capture these aspects of programming competence. In this design principle, teachers structure the class to progress from AI-assisted learning to guided practice and, ultimately, to independent performance. Assessments may require students to explain AI-generated code, justify programming decisions, debug existing solutions, document their AI interactions, and subsequently complete related tasks without AI assistance.

Traditional approaches (i.e., pencil-and-paper tests, online tests, and practical coding tasks) remain applicable for assessing conceptual and procedural knowledge. However, these approaches must be conducted within controlled classroom settings (Marco-Detchart and Lopez-Molina, 2025). In addition to traditional approaches, this study proposes assessment methods for LLM-assisted programming anchored in different theories. These methods can take oral (e.g., think-aloud protocols, code walkthroughs), written (e.g., debugging explanations, reflective reports, paper-based tests), and performance-based forms. Evaluation may also use analytic rubrics, observation checklists, and reflection guides. Teachers can also require students to submit process artifacts, such as version histories and interaction logs, to capture their reasoning and how they develop solutions (Table 2).

**Table 2:** Theoretical foundations for sample assessments of conceptual and procedural knowledge in LLM-assisted programming.

Theory	Knowledge type	What to monitor	How to assess in LLM-assisted programming	Assessment format	Assessment tools
Constructivism	Conceptual knowledge	Understanding of programming concepts (e.g., loops, algorithms, data structures, logic behind solutions)	Requires students to explain AI-generated code, predict outputs, compare alternative solutions, and justify why a solution works.	Written exam Viva voce/oral exam	Rubric for conceptual knowledge
Cognitive apprenticeship	Conceptual and procedural knowledge	Expert-like thinking process (reasoning, debugging strategies, decision-making during coding)	Use think-aloud protocols, code walkthroughs, debugging explanations, and reflection tasks showing how solutions were developed and refined.	Interviews Written (e.g., debugging explanations, reflective reports) Live coding tasks	Rubrics Observation checklist Process artifacts (e.g., code versions, prompt logs)
Self-regulated learning	Procedural knowledge	Management of programming process (planning, AI use, monitoring outputs, evaluating correctness)	Assess learning logs, prompt histories, version control records, and reflective reports documenting how AI tools were used and validated	Interviews Written (e.g., debugging explanations, reflective reports)	Rubrics Process artifacts score (e.g., code versions, prompt logs)
Social constructivism	Conceptual and procedural knowledge	Interaction with AI as scaffolding (how students negotiate, refine, and interpret AI outputs)	Evaluate prompt engineering quality, iterative refinement of AI responses, and how students integrate AI suggestions into working solutions.	Portfolio (prompt logs, reflections, annotated code)	Rubrics Portfolio score

For example, if a teacher intends to measure a student’s understanding of programming concepts, the student may be asked to explain AI-generated code, predict its outputs, and justify why a solution works. This can be done in writing (e.g., essays) or through a viva voce (oral examination). A rubric can then assess students’ conceptual understanding in this domain. This assessment approach is grounded in constructivist theory. Another example is that after students practice programming with LLM assistance, teachers may provide activities that require them to work without LLMs. Cognitive apprenticeship theory supports this assessment strategy (Collins et al., 1991), suggesting that students learn through guided participation with experts. As students acquire knowledge and skills through guided support, they can gradually demonstrate independent expertise. Recently, one study used cognitive apprenticeship theory as the foundational lens for an LLM agent’s instructional logic in a programming course (Liang, 2026). The study showed positive results in students’ programming performance. However, the study relied on a single-group pre-test-post-test design, emphasizing the need to test the proposed hypothesis in the current study.

The proposed approaches are not necessarily new (Brennan and Resnick, 2012; Gomes et al., 2016; Sun et al., 2019). These assessment formats and tools have become increasingly emphasized and necessary to align with teaching strategies and lesson designs and to support a more comprehensive assessment approach. The expected outcome is therefore a more valid representation of students’ actual programming competence. This mechanism can be empirically tested by comparing students’ AI-supported programming performance with their independent programming performance.

### 3.4 Design principle 4—embed ethical and responsible LLM use across programming pedagogy (DP4)

Excessive reliance on AI may undermine critical thinking skills (Wiederhold, 2025). Hence, teachers and students alike need to recognize the importance of responsible AI use. DP4 cuts across all components of the LAMPP model because each component raises ethical concerns. Instilling the ethical use of LLMs in programming pedagogy may not be difficult, as students and teachers share similar views on using these learning tools (Bringula et al., 2026). The literature includes many publications on ethical guidelines for using generative AI in programming (e.g., Jiao et al., 2025; Mahon et al., 2024). However, these guidelines are not anchored in educational theories, which weakens their philosophical grounding and the rationale for incorporating ethical use into programming pedagogy (Fu et al., 2025).

Ethical guidelines for LLM-assisted programming can be meaningfully framed through social constructivism. Grounded in this theory, it views academic integrity as something negotiated within the learning community rather than as a fixed set of rules. Through dialog, students, instructors, and institutions may co-construct ethical and responsible LLM use (Bringula et al., 2026). This can be achieved by jointly formulating policies and discussing acceptable and unacceptable LLM use in class and for course requirements. In this framing, joint knowledge production with LLMs promotes transparency among all parties.

From the lens of self-regulated learning, LLM use can be acceptable if students are guided to plan, monitor, and evaluate their use of these technologies during programming tasks. This is because human judgment and intervention are present throughout the programming process. For instance, planning may involve deciding when LLM feedback is appropriate or useful; monitoring involves tracking how much of the solution the student produces versus the LLM; and evaluation involves reflecting on whether the solution is correct and whether the student truly understands the code. From this perspective, these practices foster students’ responsibility to actively document their LLM use as part of the learning process.

Together, these two theoretical perspectives show that LLM-assisted programming pedagogy is both socially negotiated and individually regulated. Social constructivism highlights the need for integrity, transparency, and responsibility, while SRL emphasizes developing the internal capabilities to apply these norms in classroom activities. DP4 is justified because it translates broad ethical principles into actionable definitions and practices within a concrete educational context. It clarifies how to operationalize academic integrity and ethical LLM use in day-to-day programming pedagogy.

Drawing on these theoretical perspectives, DP4 proposes two complementary mechanisms: shared norms and self-regulated LLM use. Shared norms establish collectively negotiated expectations regarding acceptable and unacceptable uses of LLMs in programming activities. In contrast, self-regulated LLM use enables students to plan, monitor, and evaluate their use of these tools. Together, these mechanisms address the tension between the convenience of AI assistance and the need for responsible use. As discussed above, shared norms have not been widely reported as a distinct construct in programming learning (e.g., Liu et al., 2023). This study recommends that future studies identify which shared norms are agreed upon in a programming classroom setting. Thus, shared norms and self-regulated LLM use are hypothesized to be positively associated with students’ responsible AI behavior.

Appendix A provides a sample LAMPP model-guided programming activity for teachers. Teachers may use this as a template when designing course content delivery for a specific syllabus topic. They may edit the template as needed. However, Teaching, Lesson Design, Learning Engagement, Assessment, and Ethical Considerations are fixed components that must be completed accordingly. The sample lesson guide consistently lays out expectations for these components that are consistent with the model.

The sample activity is constructed for an introductory programming class. The programming problem context includes the programming task, course level, course topic, learning objective, and activity format. The topic is focused on variables, conditional statements, and loops through the task “Finding the Largest Number.” The learning objective is for students to write and explain a Python program that identifies the largest number in a list without using Python’s built-in *max()* function.

The teacher should explicitly state that they cannot use ChatGPT to generate the complete code. Instead, students must write their program first without ChatGPT's assistance. Then, they can ask it for hints. They may also ask it to explain errors during debugging. They are expected to evaluate ChatGPT’s suggestions. They revise their code based on their own reasoning and testing and must ultimately explain their final solution independently. This sequence positions the LLM as a scaffold for problem-solving.

The assessment focuses on both the final program and the process through which students develop and understand their solutions. Students will submit their initial code version, the prompts used, and brief explanations of the changes. They may also be required to provide a short explanation of what they learned and how the technology helped. An oral examination can complement these artifacts by requiring students to present their solution, explain how the code works, and predict its output. Together, these assessment components allow evaluation of both the resulting program and the student's independent understanding.

Teachers must communicate this learning process to students before the activity begins so students know the course expectations and intended outcomes. This will help establish shared class norms that emphasize equitable access, transparency, verification, and student responsibility.

## 4 Limitations

The proposed model attempts to address the challenges LLMs pose in programming education. However, the model is not a definitive solution to the complexities and nuances of these technologies. This study acknowledges the proposed model’s potential limitations. In this regard, constructivist and social constructivist strategies might overwhelm teachers and students. Therefore, a major limitation is the additional workload this may place on teachers and students. The mechanisms proposed in this paper may require more varied teaching and assessment approaches. While these approaches may provide more meaningful learning experiences and alternative evidence of student competence, they may also substantially increase teachers’ workload. For students, mechanisms under the SRL and cognitive apprenticeship theories might be overly tedious because documenting every step of their work may shift their focus from programming learning to compliance with documentation.

Beyond these theoretical limitations, practical constraints also exist. These include classroom-related factors, such as class size, teachers’ management of multiple classes, and the absence of teaching assistants. Moreover, even when teachers are willing to adopt these changes, the absence of institutional AI policies and limited teacher training may impede the implementation of the proposed design principles (Ramos et al., 2024; Villanueva et al., 2025). Another potential issue is access to AI tools. AI tools raise concerns about access to paid versions, privacy, and schools’ digital infrastructure (Jiao et al., 2025). Finally, implementing all the proposed mechanisms simultaneously may be overwhelming given the time constraints of individual courses.

## 5 Concluding remarks, recommendations, and implications for research and practice

LLMs are fundamentally reshaping programming pedagogy, creating a necessity for a modern approach to programming education and a theory-based pedagogical response to their integration. The LAMPP model addresses the potential risks LLMs pose to programming education, but its effectiveness remains to be evaluated. The model is not about LLMs per se; instead, it focuses on the pedagogical orchestration of these AI tools within programming education. Specifically, it considers how these technologies can be integrated into teaching strategies, lesson design, student engagement, assessment, and ethical practices. While the model may not capture the full complexity of the disruption these AI tools cause in programming education, it provides a structured, theory-informed foundation for understanding and guiding their integration. The future of programming education may lie not in restricting these technologies but in designing pedagogies that enable learners to collaborate with them critically, effectively, and ethically. The model may guide integration by clarifying learning goals, informing evaluation, supporting pedagogical alignment, mitigating potential risks, and encouraging rigorous, transferable educational practices.

Despite potential implementation limitations, the model provides a flexible, adaptable framework for integrating LLMs into programming pedagogy. Its design principles can be implemented incrementally and adapted to different instructional contexts, resources, and institutional conditions. Teachers should balance the teaching strategies they implement with class size and course duration. The model also provides a foundation for empirical testing and further refinement as evidence on LLM-supported programming education develops. The model proposes a shift from teacher-centered instruction to facilitated, student–LLM collaboration; from static, linear exercises to dynamic, LLM-integrated, and exploratory tasks that embed interaction with tools; from output-based evaluation (final code) to process-oriented assessment focusing on reasoning and LLM interaction; and from passive, individual task completion to active, reflective, and self-regulated learning with LLM within the boundaries of ethical use.

The DPs provide operational lenses that make the framework usable, testable, and researchable. Educational theories served as the basis for constructing the strategies; therefore, these strategies can be empirically tested to assess the effectiveness of the proposed mechanisms. From these lenses, several research implications emerge. The DPs can guide study design and may be implemented and tested across different education levels. Future studies may also investigate whether the mechanisms mitigate potential programming skill losses associated with LLM use.

To address unequal access, institutions may provide access to approved AI tools. Teachers may also design activities with clearly defined levels of permitted AI involvement: no AI tool use, in which students complete the task independently; limited AI tool assistance, in which they are permitted only for specific functions such as explanation, feedback, or debugging; and extensive AI tool assistance, in which they may be used across multiple stages of the programming process. For activities that permit extensive AI tool assistance, teachers could design tasks around features available in free versions to minimize access differences. For instance, an activity could specify the maximum number of prompts students may use.

In practical terms, the model can serve as a general guideline for redesigning curricula, classroom policies, teaching strategies, assessments, faculty development, and institutional policies. Teachers and institutions are encouraged to adopt the design principles gradually. Lastly, institutional support is also needed to develop workshops and training programs that can help educators design teaching strategies and assessment methods appropriate for tiered implementation (e.g., by class size or course), standardize templates and rubrics, and institutionalize reusable instructional resources.

## Data Availability

The original contributions presented in the study are included in the article/Supplementary material, further inquiries can be directed to the corresponding author/s.
