# Supplementary material for: Large language model-assisted modern programming pedagogy model: a perspective on teaching programming in the era of LLMs
Source: Front Artif Intell. 2026 Oct 28;9:1945247. doi: 10.3389/frai.2026.1945247 (PMC13752114; doi:10.3389/frai.2026.1945247)
Supplement: Supplementary file 1 [file Supplementary_file_1.docx]

Appendix A

A Sample LAMPP Model-Guided Activity Template for Teachers: Finding the Largest Number

**Context**

| *Programming Level:* | Introductory Programming |
| --- | --- |
| *Topic:* | Variables, conditional statements, and loops |
| *Activity Name:* | Finding the Largest Number |
| *Learning Objective:* | Students should be able to write and explain a Python program that finds the largest number in a list. |
| *LLM Version:* | Use the free version of ChatGPT. |
| *Activity Format:* | Individual activity; ChatGPT serves as a coding assistant. |

**Teaching** (*Describe the role and limits of LLM use.*)

- The teacher allows students to use an LLM as a coding assistant for hints.
- The outright generation of complete code using ChatGPT is prohibited.

**Lesson Design** (*Describe the activity and expected outcomes.*)

- Write a Python program that accepts a list of numbers and finds the largest number without using Python’s built-in function.

**Learning engagement** (*Describe the activity procedure.*)

1. Conduct the activity during the laboratory session.
2. Only the free version of ChatGPT will be allowed.
3. Students write their program first, without ChatGPT’s help.
4. Ask ChatGPT for a hint if they are stuck.
5. Ask for an explanation of an error after testing their code.
6. Evaluate and revise ChatGPT’s suggestions.
7. Explain their final solution independently.

**Assessment** (*Specify the assessment components and requirements.*)

| **Component** | **What to Comply With? (One or combination)** |
| --- | --- |
| Self-reflection | A short explanation of what they learned and how ChatGPT helped. |
| Initial solution (process artifact) | The original first version of the code. |
| AI interaction (process artifact) | Prompts used |
| Revisions (process artifact) | Brief explanation of what they changed and why |
| Oral examination | Presentation, code explanation, output prediction |

**Ethical Considerations** (*Specify the ethical requirements for LLM use.*)

- Only the free version of ChatGPT is allowed to promote equitable access.
- The activity will be conducted in the laboratory to ensure equitable access to the LLM.
- Students should disclose how they use the LLM.
- Students should not submit LLM-assisted code that they cannot explain.
- Students should verify the LLM’s suggestions through testing and reasoning.
- Students remain responsible for the correctness of their submitted programs.
